# Effectiveness of interventions to optimise dietary intakes in the first 1000 d of life in Indigenous children: a systematic review

**DOI:** 10.1017/S1368980021004328

**Published:** 2022-02

**Authors:** Oyepeju Onifade, Lucy Kocanda, Tracy Schumacher, Megan Rollo, Kym Rae, Kirsty G Pringle

**Affiliations:** 1 School of Medicine and Public Health, University of Newcastle, Callaghan, NSW, Australia; 2 Pregnancy and Reproduction Program, Hunter Medical Research Institute, Lot 1 Kookaburra Cct, New Lambton Heights, NSW 2305, Australia; 3 Department of Rural Health, University of Newcastle, Tamworth, NSW, Australia; 4 Priority Research Centre for Health Behaviours, University of Newcastle, Callaghan, NSW, Australia; 5 School of Health Sciences, University of Newcastle, Callaghan, NSW, Australia; 6 Priority Research Centre for Physical Activity and Nutrition, University of Newcastle, Callaghan, NSW, Australia; 7 Mater Research Institute, Aubigny Place, South Brisbane, QLD, Australia; 8 University of Queensland, Faculty of Medicine, Herston, QLD, Australia; 9 School of Biomedical Science and Pharmacy, University of Newcastle, Callaghan, NSW, Australia

**Keywords:** Indigenous, Nutrition intervention, Nutrition, Co-design, Participatory research, First 1000 d, Child nutrition

## Abstract

**Objective:**

Indigenous infants are disproportionately more likely to have negative outcomes compared to non-Indigenous infants with suboptimal nutrition in the first 1000 d playing a major role. This review aimed to systematically assess the effectiveness of interventions designed to optimise dietary intake and/or nutrition-related behaviours among Indigenous infants globally and to identify whether Indigenous populations were involved in the co-design of the intervention.

**Design:**

Articles published before June 2020 that reported nutrition-related interventions and outcomes for Indigenous infants were identified from a database search. Data extracted included study aims and design, target population, geographical location, the health condition of the participants, intervention characteristics and outcomes. A narrative synthesis consisting of effects and acceptability of the interventions and involvement of participants in the study design were highlighted.

**Settings:**

Population-based intervention studies that focused on improving dietary intakes and/or nutrition-related behaviours of Indigenous infants in the first 1000 d of life were included in this review.

**Results:**

Of the 2784 studies identified, three studies met the inclusion criteria. These were conducted among two Indigenous tribes in Guatemala and the USA. Two studies reported the food and nutrient intake of participants with one study showing an improvement in dietary intake of the infants. Only one study reported community participation in the study design, intervention design and implementation, and acceptability of the intervention by the participants.

**Conclusion:**

Engaging Indigenous communities throughout the entire process of nutrition interventions could have beneficial effects through improved outcomes in the first 1000 d of life.

Globally, Indigenous peoples are population groups with complex longitudinal histories^(1)^. Indigenous nations are diverse and comprise people who share a common culture, heritage, language, geography and a desire for common interaction^(2)^. According to the UN, it is estimated that there are at least 370 million Indigenous people belonging to 5000 Indigenous groups in more than 70 countries^(3,4)^. Approximately 5–16 % of the world’s population identify as Indigenous^(5)^. Although these populations have histories that date back thousands of years, some histories have been better documented than others^(6)^. Many Indigenous populations have experienced a continuous cycle of historical trauma with pre-invasion and pre-colonial societies that developed on their territories^(2,4)^.

Pre-colonial health, nutrition and well-being of Indigenous populations are not well documented. However, studies have shown that most Indigenous groups were able to control diseases and enjoy higher levels of overall health and wellness prior to their colonisation^(7)^. The impact of colonisation on Indigenous people is long-lasting and continues to affect physical, social and health outcomes^(3)^. The pre-colonial lifestyle of many Indigenous peoples involved high levels of physical activity and sophisticated agricultural and aquacultural practices^(8)^. Cultural practices highlight that Indigenous people’s diet consisted of a high intake of nutrient-dense foods that were generally low in sugar^(9,10)^.

Optimal infant nutrition in the first 1000 d is important in order to achieve favourable health outcomes for all populations^(11)^. Studies have shown that several factors influence the current dietary patterns of Indigenous children in the first 1000 d of life including geographic location, rural lifestyles, ethnicity or Indigenous identity, dispossession from land, and environmental factors^(7,12,13)^. Early childhood nutrition, beginning from birth through infancy includes breastmilk, infant formula and complementary feeding^(14)^. Australian Aboriginal children were traditionally breastfed until the age of 3 to 4 years, usually fed on demand, and weaning age often depended on the arrival of a younger sibling^(13)^. Similarly, in Canadian Indigenous populations children were breastfed for many years, and breast-feeding knowledge was handed down from older community members and elders to mothers^(15)^. Breast-feeding duration in both countries has however declined in recent times^(16,17)^.

Improved complementary feeding alongside breast-feeding has been identified as the major intervention necessary to improve child health outcomes^(18)^. After 6 months, breastmilk alone is no longer sufficient to meet the increased nutritional demand of the infant, thus the need to introduce adequate complementary foods^(19)^. Complementary feeding practices among Indigenous populations vary across different countries and regions and findings have shown the likely need for a general improvement in feeding practices in order to provide optimal nutrition^(20)^. There is little evidence available on the complementary feeding practices of Indigenous populations in the pre-colonial era; however, studies have shown that traditional practices among Aboriginal Australians was to await demand of family foods from infants before shifting to this mode of feeding^(21)^. Indigenous traditional complementary feeding practices have changed over time with market foods being integrated into communities, and substitutes being more expensive and less nutritious than traditional foods. These post-colonial changes have generally occurred out of necessity to replace foods that have become unavailable due to removal from traditional lands following colonisation. These removals resulted in a loss of hunting grounds, including fish/marine animals in rivers, lakes and sea as well as loss of other food sources and meant that Indigenous people were then dependent on poor-quality foods provided by those settings^(20)^. Individual choices of parents influence this change with preparation of market foods becoming more convenient^(20)^. Evidence has shown both early and late introduction of complementary foods among Indigenous infants with different geographical and cultural characteristics^(20)^. A study conducted among Canadian First Nations people showed that 19 % of infants received solid foods before 2 months of age and 57 % before 4 months of age^(22)^. A similar study among the Thailand Karen tribe showed that complementary foods were introduced to infants at approximately 3 months^(23)^. Evidence from the Ingano people of Columbia showed that almost all infants had complementary food at 4 months of age^(24)^. However, a study conducted among the Indian Bhils tribe reported that the majority of infants received complementary foods after 6 months of age with only 1 % receiving complementary foods between 4 and 6 months of age^(25)^. Similar studies have also shown that among the Awajun people of Peru, more than half of the infants were exclusively breastfed at 6 months^(26)^.

Interventions designed to optimise dietary intakes in Indigenous populations require a combination of Indigenous ways of knowing and evidence-based strategies^(27)^. Evidence has shown that a participatory approach and community engagement are important factors required in intervention studies among Indigenous populations^(28)^. A study conducted among Indigenous Australian women to improve nutrition concluded that factors, such as understanding community needs, understanding the impact of historical factors on health, consideration of family and community tensions, and promotion of community determination towards improvement through engagement of long-term partnerships, all need to be considered in developing nutritional interventions^(29)^. Furthermore, this study showed that a community voice helped to develop a frame of reference for their health evidence base. According to a systematic review by Ashman *et al.*, community collaboration in study designs and delivery of interventions by Indigenous workers improved nutrition-related outcomes in pregnancy, including improved birth weights, and breast-feeding initiation and duration^(30)^. A similar review by Gwynn *et al.* showed that adopting an ecological approach is important in nutrition interventions among Indigenous populations^(31)^. The review showed that strong community engagement influenced a wider range of outcomes compared with others with less engagements.

This review aimed to systematically assess the effectiveness of interventions designed to optimise dietary intake and/or nutrition-related behaviours of Indigenous children globally in the first 1000 d of life. Furthermore, it aimed to identify whether Indigenous populations were involved in co-designing the interventions and, if so, how they were involved. Factors influencing the dietary patterns of Indigenous children and the effects of these factors on their health outcomes were also assessed in this review. Findings from this review will improve knowledge on the importance of community collaboration and inform future nutrition intervention studies among Indigenous populations.

## Methods

The Preferred Reporting Items for Systematic Reviews and Meta-Analyses (PRISMA) statement was adhered to in designing and preparing the manuscript for this study. The protocol for this systematic review was registered with the online PROSPERO database (University of York centre for reviews and Dissemination) (CRD42020154552).

### Study identification

Studies published in English prior to 26 July 2019 from relevant electronic databases (MEDLINE, Cochrane, EMBASE, CINAHL, EMcare, Maternal and Infant Care) were gathered using identified keywords and index terms. A rerun of the search was conducted on 22 May 2020. The search terms were divided into four groups:

#### Indigenous key terms

Aborigin* or inuit* or torres strait* or indigenous or ainu or sami or san or first nation* or maori*.mp or exp african continental ancestry group/or exp american native continental ancestry group/or exp asian continental ancestry group/or oceanic ancestry group/or inuits/or exp population groups or native or people* or population* or alaska*mp..

#### Nutrition key terms

Diet/or Weight Loss/or Nutritional Status/or nutrition assessment/or Feeding Behavior/or Food Preferences/or Infant Nutritional Physiological Phenomena/or Infant Food/or Energy Intake/or Child Nutrition Sciences/or Nutrition Disorders/or nutrition* or diet* adj2 (intervention* or intake or habit* or deficien*.mp or eat* or feed* or diet* or behavio* or pattern*.mp. or food choice*.

#### Child and infant key terms

Child/or infant* or baby or babies or newborn or toddler* or ‘first 1000 d’. mp., or Infant, Very Low Birth Weight/or Infant, Low Birth Weight.

#### Complementary feeding key terms

Weaning/or wean*.mp. or complementary feed*.mp. or first foods.mp. or introduction to solids.mp.

The Boolean phrase AND was used between groups and OR within groups.

### Eligibility criteria

Inclusion and exclusion criteria were developed using the PICOS (population, intervention, comparator and outcome) tool^(32)^ and are described below.

### Participants/population

Studies were included where the target population were Indigenous children in the first 1000 d after birth. Indigenous peoples were defined as communities that live within, or are attached to, geographically distinct traditional habitats or ancestral territories. They identify themselves as being part of a unique cultural group and descendants from groups present in the area before modern states were created and current borders were defined^(33)^. All international Indigenous populations were included in the study. The children in the included studies had at least one biological parent with Indigenous heritage. Studies conducted across various population groups but involving Indigenous groups were included, provided the data from the Indigenous population groups were analysed separately and reported as such.

Studies were included if, at the time of the study, all children were non-hospitalised, in their family or community environment and without a specific disease. Studies conducted on children with malnourishment and failure to thrive were included.

### Types of studies

Studies with any intervention component aimed at first foods, optimising food and/or nutrient intake and/or nutrition-related behaviour during, and/or following weaning were included in this review. Any intervention study designs including randomised controlled trials and quasi-experimental studies (non-randomised control trials and pre- and post-test studies) were included. There were no limitations to the mode of delivery of the intervention, and included intervention targeted directly at families or at the population/community level. No qualitative studies were included in the review. Protocols for ongoing studies with no evaluation outcomes reported, grey literature, theses, and conference abstracts, were excluded from this review.

### Comparators

Studies with a comparator group (e.g. a similar group in the same population receiving no intervention, another population group or Indigenous community, or a pre-/post-test study design) were included in this review.

### Outcome measures

Studies were included that reported any of the following outcome measures: food and/or nutrient intake and/or nutrition-related behaviours such as modification of dietary intake, relative to age and as a result of a nutritional intervention; health and/or nutritional status of child (e.g. growth); how community involvement in the design of the intervention influenced dietary intake; and factors influencing dietary intake and/or nutrition-related behaviours.

### Study selection

All studies identified by the databases were retrieved and exported into Covidence^(34)^, an online tool for study selection and data extraction, and duplicates were removed. The first phase of study identification was conducted by two independent reviewers (OO and LK) who assessed the relevance of each study by title and abstract against the inclusion criteria to determine the need for full-text review. If either reviewer identified a potentially eligible study for full-text extraction, the full text was retrieved. The second phase of study identification was an assessment of full-text review of retrieved studies, completed by two independent reviewers (OO and LK) to determine if they met the inclusion and exclusion criteria. In cases where reviewers disagreed, the third reviewer (KP) was consulted to determine if the study met the inclusion criteria. Reference lists of included studies were hand-searched to identify additional studies that met the inclusion criteria.

### Study quality

Of the several tools available for quality assessment in systematic reviews, no tool considered community co-design/engagement/leadership in Indigenous populations^(35)^. Therefore, we have only reported on whether the studies involved the community in any phase of the study, from design to delivery of the intervention, and the nature of this involvement. Articles meeting the inclusion criteria were assessed for methodological quality using the Academy of Nutrition and Dietetics Quality Criteria Checklist for Primary Research Standardized tool^(36)^. This tool, used to assess quality in nutrition reviews, has been used in previous reviews on Indigenous populations^(30,37)^. This tool consisted of ten criteria that assessed the strength of the research design, relevance and validity. Items assessed included sample selection method, methods of controlling for confounding factors, reliability of outcome measures and statistical analysis. Two reviewers independently used the checklist to rate the overall quality of studies as positive, neutral or negative (OO and LK). Disagreements regarding study quality between the two reviewers were discussed, and a verdict was determined by the third reviewer (KP).

### Data extraction and synthesis

Data extraction was conducted by one reviewer (OO) and cross-checked by the second independent reviewer (LK) for accuracy and consistency. Key data reported included participant information, study design, intervention description and delivery, community involvement in design and intervention, outcomes, comparison group, study quality and key findings. Due to the heterogeneity of the studies included, a meta-analysis could not be conducted. Thus, the effectiveness of nutrition-specific interventions on health outcomes of Indigenous infants in the first 1000 d was described in a narrative synthesis. A structured summary, effects and acceptability of interventions and involvement of participants in study design were highlighted in the data synthesis.

## Results

### Study selection

**Fig. 1:**
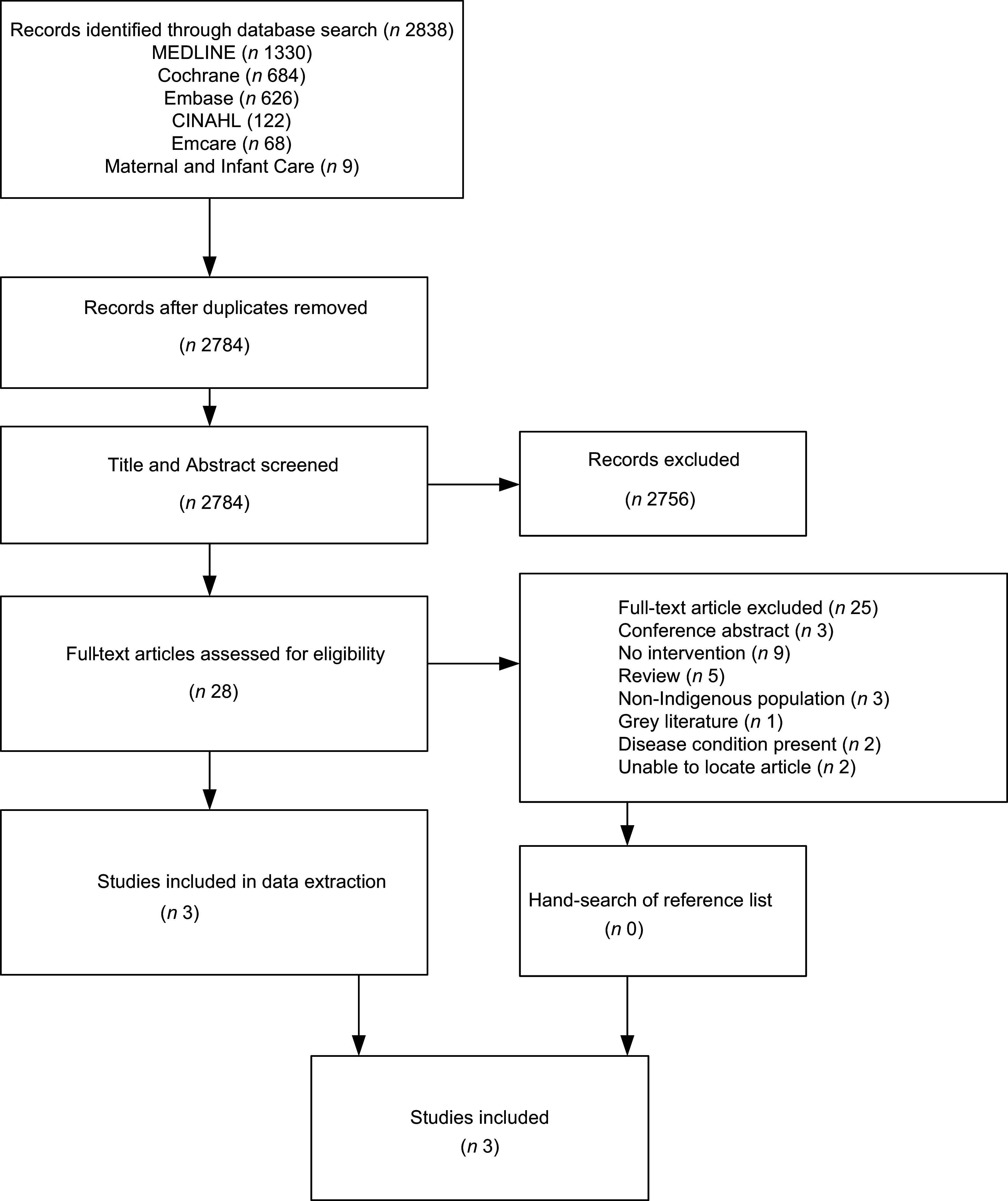
PRISMA flow diagram. PRISMA, The Preferred Reporting Items for Systematic Reviews and Meta-Analyses

A total of 2838 articles were identified from the initial database search with 2784 articles identified after duplicates were removed (Fig. 1). During title and abstract screening, 2756 records were excluded. The remaining 28 full-text records were screened, and a further 25 articles were excluded. Three articles remained and were included in this study. No additional records were identified by hand-searching the reference lists of included articles.

### Characteristics of included studies

All studies included in this review were published between 2010 and 2018^(38–40)^. The sample sizes of the included studies ranged from 94 to 324, with infant ages ranging from 6 to 36 months (Table 1). Two of the studies were conducted among American Indigenous populations^(38,39)^, while the third study was conducted among a Guatemalan Indigenous Mayan population^(40)^ (Table 1). The interventions ranged in duration from 6 to 24 months and the number of exposures to the interventions ranged from 6 to 21.

**Table 1:** Characteristics of included studies

Reference	Location	Aims	Study design	Participant settings	Inclusion/exclusion criteria	Sample size	Health condition of participants	Age of participants	Comparators
Martinez *et al*. 2018^(40)^	Tecpan municipality, Guatemala	To examine the impact of an intensive, individualised approach to complementary feeding education for caregivers on feeding practices and growth of stunted infants over usual care.	Single centred, individually randomised RCT parallel group superiority trial.	Kaqchikel Maya tribes in rural Guatemala.	Inclusion: Children aged 6–24 months; LAZ/HAZ ≤ –2·5 sd on WHO growth standards. Exclusion: acute malnutrition (LAZ/HAZ ≤ –2 SD); severe medical illness.	324 children (total); Intervention: *n* 161; Control *n* 163 (usual care).	Malnutrition	Intervention Maternal age: 26·8 ± 7·0 years; Child age: 15·8 ± 5·2 months; Control Maternal age: 27·4 ± 6·6 years; Child age: 15·1 ± 5·2 months.	Usual care: children aged 6–24 months; LAZ/HAZ: ≤–2·5 SD on WHO growth standards.
Karanja *et al*. 2010^(39)^	Idaho, Oregon, Washington, USA	1. Determine the feasibility of delivering community-wide interventions alone, or in combination with family-based interventions, to promote breast-feeding and reduce the consumption of sugar-sweetened beverages in an obese community 2. Determine if the interventions decrease BMI *Z*-scores in children aged 18–24 months	Pre-/post-study design.	Three American Indian tribes. Northwest Portland Area Indian Health Board.	Expectant mothers and their families	Total enrolled in study: *n* 205; Tribe A: *n* 63, B: *n* 62 and C: *n* 80; Intervention alone: *n* 63; Intervention with family component: *n* 142.	None	Intervention: maternal age: 25·0 ± 5·8 years; Child age: 18–24 months.	Cohort of children born 2 years earlier to the same tribes. Where data were unavailable from this cohort of children, data were compared to Paediatric Nutrition Surveillance System (PedNSS). Each study child was paired with a PedNSS child and matched by sex, birth month and age at 18–24 months measurement point to adjust for disparity in *Z*-scores.
Hoffhines *et al*. 2014^(38)^	Oklahoma, USA	Assessment of prevailing infant and toddler feeding practices to develop a future maternal education plan.	Non-randomised control trial	American Indian tribes represented by the Southwest Intertribal Health Board.	Mothers of toddlers with tribal affiliation aged 12 to 36 months. Siblings of toddlers could be included.	*n* 94	None	Maternal age: 13–39 years; Child age: 12–36 months.	None

RCT, randomised control trial; LAZ, length-for-age *Z*-score; HAZ, height-for-age *Z*-score.

The study by Martinez *et al.* was a randomised control trial among a Guatemalan Indigenous Mayan population to improve the stunted growth status of the children. The intervention explored the impact of intensive individualised complementary feeding education for caregivers^(40)^. Karanja *et al.* conducted a pre- and post-test study among three American Indian/Alaskan Native populations^(39)^. The study aimed to prevent overweight and obesity in children, and it showed prevailing levels of overweight in the communities studied prior to intervention. The intervention assessed the feasibility of delivering a community-wide intervention, alone or in combination with a family-based intervention^(39)^. The study design was not reported by Hoffhines *et al.*; however, it was categorised as a non-randomised control trial from the description provided. The study aimed to prevent overweight and obesity in children; however, these were not assessed at baseline. It recruited participants from a single American Indian population. The intervention assessed the implementation of a maternal education programme to improve feeding practices^(38)^.

Details of the study interventions are described in Table 2. Two studies^(39,40)^ delivered the intervention through home visits, while Hoffhines *et al.* delivered the intervention through face-to-face visits at local paediatric clinics. Hoffhines *et al.* included media communication as a mode of delivery.

**Table 2:** Intervention features

References	Type of intervention	Duration of intervention	Mode of delivery	Number of exposures	Follow-up interval	Intervention features (development and content)
Martinez *et al*. 2018^(40)^	Individualised complementary feeding education	Intervention: 6 months	Intervention: home visits	Intervention: 6	Intervention: 30 d	Intervention: Provision of multiple micronutrient powder supplement; Biweekly food rations; Complementary feeding based on WHO’s recommendations; Open-ended questions to explore caregiver’s perception of child’s dietary adequacy; Highlight positive dietary changes since preceding visit; Set concrete goals for subsequent visit.
		Usual care: 6 months	Usual care: home visits	Usual care: 6	Usual care: 30 d	Usual care: Growth monitoring; Provision of multiple micronutrient powder supplement; Biweekly food rations; Complementary feeding based on WHO’s recommendations
Karanja *et al.* 2010^(39)^	1. Community-wide intervention 2. Community-wide intervention containing a family component	6-month cycles	Media based- brochures, flyers, videos, newspaper articles and home visits	Community-wide: not stated	Community-wide: not stated	Community-wide: Raising awareness; providing health education; facilitating individual behaviour change; augmenting public health practice; and modifying environments and/or policies related to breast-feeding, sugar-sweetened beverages and water consumption.
				Family interventions: 7 to 21	Family interventions: not stated	Interventions include: 1.)Increase breast-feeding initiation and duration; 2.)Limit introduction of sugar-sweetened beverages to infants and toddlers; 3.)Promote consumption of water for thirst among toddlers. Family interventions: Eight visit clusters with each cluster having up to three distinct contacts. Only one required to be a face-to-face while the other two could be by phone. Cluster 1–3: Client-specific plan for initiating and maintaining breast-feeding created; Cluster 4–7: Intervention implementation and monitoring; Cluster 8: Closure activities; Cluster 1 occurred before baby’s birth while clusters 2–4 occurred within 0–3 months of birth.
Hoffhines *et al*. 2014^(38)^	Group education undertaken during pregnancy and educational follow-up postpartum	24 months	Face-to-face group sessions	One education session in pregnancy (gestational age not specified); Nine postpartum sessions (2 and 6 weeks; 4, 6, 9, 12, 15 18 and 24 months).	2 to 12 weeks	Certified lactation specialist conducted class for pregnant mothers on benefits of breast-feeding, breast-feeding myths and problem-solving, latching techniques, breast-feeding positions, use of breast pumps and healthy infant feeding practices. Educational follow-up and support. Nutritional guidance for mothers during visits.

### Description of nutrition and nutrition-related behaviour interventions

The nutrition intervention in the studies varied from nutrient supplementation (Martinez *et al.*) to improving breast-feeding practices and water intake (Karanja *et al.* and Hoffhines *et al.*). The study by Martinez *et al.* included a daily micronutrient powder supplement (Chispitas/sprinkles, daily dose composition: ferrous fumarate 12·5 mg, zinc gluconate 5 mg, retinol acetate 300 μg, folic acid 160 mg and ascorbic acid 30 mg), a biweekly protein-rich food ration (beans 1000 g, 20 eggs and 900 g of Incaprina, a soya-based complementary food supplement) and messages about complementary feeding based on WHO recommendations (continued breast-feeding on demand, appropriate consistency of complementary foods, appropriate age-adjusted meal frequency and provision of a diversity of foods) for their target population of Mayan women (Table 2). Conversely, of the other two studies which focused on improving breast-feeding practices and water intake^(38,39)^, Karanja *et al.* focused mainly on increasing breast-feeding practices, while Hoffhines *et al.* educated mothers on breast-feeding techniques and healthy feeding. Furthermore, Karanja *et al.* focused on delaying the introduction of sugar and sweetened beverages and promoting water consumption for thirst among toddlers (Table 2). Details about the components of the healthy feeding education package were not provided in the Hoffhines *et al.* study.

### Outcomes

#### Food, nutrient intake and nutritional status following intervention

Two of the three studies^(38,40)^ reported the food and nutrient intake of participants. The study by Hoffhines *et al.* focused on the nutrient intake of participants, while Martinez *et al.* focused on food intake.

Following the intervention by Hoffhines *et al.*, which aimed to prevent obesity, the energy intake of participants was lower at the 5th–10th percentile and higher at the 25th–95th percentile than the comparable percentiles in the estimated energy requirement (Table 3). Thus, the intervention was only partially successful. Carbohydrate and protein intakes were higher than the estimated average requirement in both the intervention and non-intervention groups, while fibre intake was less than the recommended adequate intake and failed to improve with intervention (Table 3). The study also showed that intake of water-soluble vitamins and minerals, such as Zn, Fe, Ca, P and Mg, were above the recommendations^(38)^; however, this contrasts with some of the finding in the Hoffhines *et al.* study (Table 3). For micronutrient intake, Hoffhines *et al.* showed that the infants had an adequate intake of vitamin A, a notably low intake of vitamin D and a low intake of vitamin E compared to the recommended standard.

**Table 3:** Outcome measures

Reference	Primary outcome(s)	Secondary outcome(s)	Outcome measures	Results	Conclusion
Martinez *et al*. 2018^(40)^	Growth: change in length/height-for-age *Z*-score (LAZ/HAZ)	Feeding indicator: minimum dietary diversity; Minimal meal frequency; Minimal acceptable diet.	Weight Length/height Minimum dietary diversity: food from ≥ 4 food groups. Minimum meal frequency: breastfed children: ≥ 2 solid, semi-solid or soft foods; ≥ 3 solid, semi-solid or soft foods. Non-breastfed children: ≥ 4 solid, semi-solid, or soft foods or milk feeds. Minimum acceptable diet: meeting both minimum dietary diversity and minimum meal frequency.	Primary (growth) outcome: • No change in LAZ/HAZ at 6 months (LAZ/HAZ change difference 0·07 (95 % CI 0·04, 0·18)). • Weight-for-age *Z*-score (WAZ) and weight-for-length (WLZ)/weight-for-height (WHZ) declined over 6 months in both study arms, resulting in a non-significant trend favouring the intervention arm (WAZ change difference 0·08 (95 % CI –0·02, 0·19); WLZ/WHZ change difference 0·08 (95 % CI –0·08, 0·24)). Secondary (feeding indicator) outcome: • 22 % improvement in minimum dietary diversity in the intervention (RR 1·22, 95 % CI 1·11, 1·35) with an absolute difference of 16·9 % (95 % CI 8·9, 25·0). • 23 % improvement in minimal acceptable diet in the intervention (RR 1·23, 95 % CI 1·08, 1·40) with an absolute difference of 15·9 % (95 % CI 6·4, 25·5). No significant improvement in minimum meal frequency (RR 1·02, 95 % CI 0·94, 1·12). Linear regression: • Female sex predicted improved growth (LAH/HAZ) at 6 months of 0·30 sd (95 % CI 0·15, 0·45; *P* < 0·0001). • Positive point change in family poverty score predicted improvement in LAH/HAZ at 6 months of 0·01 sd (95 % CI 0·001, 0·016; *P* = 0·02). • Increase in household number of < 5 children not significant with worse growth (change in LAZ/HAZ of 0·12 (95 % CI –0·29, 0·04; *P* = 0·14) for 2 children, and –0·24 (95 % CI –0·49, 0·002; *P* = 0·05) sd for 3 or more children.	1. Nutrition education can result in significant improvements in child growth following early childhood stunting. 2. Infant feeding and use of food rations can be improved through enhanced caregiver dietary education. 3. Enhanced education acted independently of food supplementation to improve utilisation by caregivers.
Karanja *et al.* 2010^(39)^	Change in *Z*-scores (for age) of BMI	Feasibility of intervention	• Anthropometry • • Breast-feeding status • • Confidence to implement study goals	Anthropometric: • BMI *Z*-scores decreased by 0·75 in family intervention group. • Height and weight were not statistically different between the three tribes. • AI children appear to grow faster than WHO reference as children gained more weight, height and BMI over the first 6–9 months of life. Breast-feeding: Breast-feeding initiation and 6 months rates were 14 % and 15 % higher than national PedNSS data for AI/AN, respectively. Sugar-sweetened beverage consumption: 60 % of participants expressed a high level of confidence to limit sugar-sweetened beverages for the family, serve water as primary beverage at meals and when thirsty. Quantitative measures of sweetened beverages consumed were not obtained.	Simple interventions may slow trend in escalating overweight and obesity in children. Interventions to prevent excess adiposity in infants and toddlers are both feasible and acceptable to the AI/AN tribes.
Hoffhines *et al.* 2014^(38)^	Macro- and micronutrient intake Breast-feeding		Nutrient intake in past 24 h Breast-feeding initiation, duration and termination	Estimated energy requirements (EER): • Infant energy intake at 5th and 10th percentile was lower than EER. • Energy intake at 25th and 95th percentile was higher than EER. Carbohydrate: • Carbohydrate and protein intake of infants was higher than estimated average requirements (EAR). • Carbohydrate intake was exceeded by 66·8 g and 51·2 g, and protein by 36·8 g and 32·8 g in cohort and survey groups, respectively. Fibre: • Fibre intake was much less than the recommended adequate intake. Toddlers consumed 42 % of recommendation. Fat: • High-fat meat products were items of choice among toddlers. • 60 % of 15 to 18 months olds, 56 % of 19 to 24 months olds and 36 % of 12 to 14 months olds consumed at least a high-fat meat product daily. Fat-soluble vitamins: • Adequate intake of vitamin A (96 % and 155 % of recommended intake in control and intervention groups). • Notable deficiency for vitamin D (88 %and 79 % of recommendation). • Low intake of vitamin E compared to the recommended standard (48 % and 56·8 % of recommendation). • 35 % met recommended intake of vitamin K. Water-soluble vitamins: • Toddlers consumed above recommendation for thiamine, riboflavin, niacin, vitamin B_6_, folate, vitamin B_12_ and vitamin C. Minerals: • Survey and intervention groups exceeded recommended intakes for Fe (332 % and 160 %), Zn (251 % and 220 %), Ca (130 % and 133 %), P (223 % and 180 %) and Mg (218 % and 173 %), respectively. Breast-feeding: • Initiation rates increased to 89 % from 59 %. • Sustained at 35 % and 12 % at 6 and 12 months, respectively.	Despite intervention energy intake significantly exceeded amount required for normal weight gain and growth. Food choices remained high in sugar persisted despite education. Low fibre intake was attributed to low intake of fruits, vegetables and whole grains. High-fat meat products were frequent items of choice.

AI, American Indian; AN, Alaskan Native; LAZ/HAZ, length/height-for-age *Z*-score; RR, relative risk; WAZ, weight-for-age *Z*-score; WHZ, weight-for-height *Z*-score.

The intervention by Martinez *et al.* also reported the food intake of infants. The study showed an improvement in the proportion of children achieving the minimum dietary diversity, measured as a result of receiving food from four or more food groups^(40)^. It also reported an improvement in the proportion of children achieving the minimal acceptable diet, measured as meeting both minimum dietary diversity and minimum meal frequency during the previous day^(40)^. The study showed no change in length-for-age *Z*-scores at 6 months and a decline in weight-for-age and weight-for-length *Z*-scores over 6 months^(40)^; however, decline in weight was less in the control arm (Table 3).

In contrast to the study by Martinez, the study by Karanja *et al.* showed that following intervention, there was a rapid increase in weight, height and BMI over the first 6–9 months of life among all infants in the two intervention groups in comparison to the WHO reference standards^(39)^. This was a negative outcome as reported by the authors, as rapid BMI increase was expected to have been slower than observed in the children in the study, although the children were still within the normal range. The study compared community-wide-based interventions with or without a family-based component and showed that despite the high BMI in all groups, BMI scores among infants in the group with a family component decreased by 0·75 units compared to the other groups. There was no statistical difference after intervention between the tribes.

#### Community involvement, design and acceptability of the interventions

All included studies reported the design of their intervention. The interventions for the American Indian women and the Mayan women were delivered by community health workers^(39,40)^, while the intervention for the American Indian/Alaskan Native women was delivered by a certified lactation specialist^(38)^. The Indigenous status of the health workers and staff who delivered the intervention was not specified in any of the three studies. The intervention was delivered through home visits to the American Indians and Mayan infants and their mothers^(39,40)^, while delivery of the intervention to the American Indian/Alaskan Native infants and their mothers was at local paediatric clinics.

Community involvement in the design of the intervention was only reported by Karanja *et al.* in the study for the American Indian/Alaskan Native population^(39)^. This study conducted focus groups and interviews at the beginning of the project, which informed the design of the intervention. The study ensured that the structure of the intervention was designed to fit the specific needs of the participants using a home-visiting model of eight-visit clusters.

The acceptability of the intervention was only evaluated in the study of the American Indian/Alaskan Native women^(39)^. The authors reported that the intervention was acceptable to participants. Confidence to implement study goals was used as an aspect of the acceptability of the intervention. This was assessed by the parents/guardians indicating their confidence and rating the usefulness of the intervention in encouraging them to achieve the goals using a five-point scale ranging from ‘1-strongly disagree to 5-strongly agree’, for a number of statements related to their intervention knowledge. Parents expressed a high confidence with a score of 4 or above.

#### Quality assessment

Of the three studies included in this review, only one^(40)^ was classified as a high-quality methodological study (Table 4). One study was classified of a neutral quality^(39)^, while the third was of poor quality^(38)^. Two of the three included studies clearly stated their research questions^(39,40)^, and the inclusion/exclusion criteria were clearly described in two of the studies^(38,40)^. All studies specified the age range of the participants (Table 4). Two of the studies did not clearly specify how the participants were selected^(38,39)^, and it was therefore challenging to ascertain how participants were distributed into groups in these studies. Furthermore, Karanja *et al.* did not adequately describe the health and demographic characteristics of the participants.

**Table 4:** Study quality based on Academy of Nutrition and Dietetics checklist

	Martinez *et al*. 2018	Karanja *et al.* 2010	Hoffhines *et al.* 2014
Was the research question clearly stated?	Yes	Yes	Unclear
Was the selection of study subjects/patients free from bias?	Yes	Unclear	Unclear
Were study groups comparable?	Yes	Yes	No
Was method of handling withdrawals described?	No	No	No
Was blinding used to prevent introduction of bias?	Yes	No	No
Were intervention/therapeutic regimens/exposure factor or procedure and any comparison(s) described in detail? Were intervening factors described?	Yes	Yes	No
Were outcomes clearly defined and the measurements valid and reliable?	Yes	Yes	No
Was the statistical analysis appropriate for the study design and type of outcome indicators?	Yes	Yes	No
Are conclusions supported by results with biases and limitations taken into consideration?	Yes	No	No
I Is bias due to study’s funding or sponsorship unlikely?	No	No	No
Overall rating	Positive	Neutral	Negative

## Discussion

This review synthesised evidence from studies that investigated the effectiveness of nutrition-specific interventions on nutrition and growth outcomes of Indigenous children in the first 1000 d of life. Community involvement in the design and implementation of the intervention was reviewed. The three included studies were conducted among two Indigenous populations in the USA and Guatemala. This review found that nutrition-specific interventions targeted towards improving the nutrition and health of Indigenous children aged 6–36 months appear to be effective. Although only one study^(39)^ involved community consultation in the design and implementation of the intervention, this community consultation was reported by the authors to be effective. The review also found that only one of the studies was of high quality.

Findings from this review suggest that nutritional interventions such as food fortification, maternal education, improved breast-feeding practices and appropriate complementary feeding could improve the nutritional status of Indigenous children. Food fortification has been found to be effective when accompanied by a balanced approach to accessibility, affordability and availability for the population in need^(41)^. A similar review on maternal and child nutritional interventions globally revealed that counselling on breast-feeding and fortification with vitamin C, vitamin A and Zn have the greatest potential in reducing child morbidity^(42)^. Further, the review by Bhutta *et al.* also reported that improving complementary feeding through strategies such as nutritional counselling and food supplements could substantially reduce stunting-related burden of disease^(42)^. Improved feeding practices are an important component of infant nutrition. Two included studies in the present review involved interventions focusing on improving breast-feeding practices and showed that an improvement in breast-feeding practices could lead to a substantial change in anthropometric measurements^(38,39)^. The third study focused on improving complementary feeding and also showed significant improvements in anthropometric measurements^(40)^. Only one study in this review reported factors that likely influenced infant feeding^(38)^. This study showed that young maternal age, a lack of resources for lactation education and maternal support during breast-feeding continuation could influence breast-feeding practices in their study population. Further, similar to previous studies^(43,44)^, this study showed that the proximity to convenience stores and availability of food stamps influenced the purchase of sugar-sweetened beverages.

Implementing effective nutrition promotion strategies during early childhood could help reduce the burden of intergenerational exposures to disease among Indigenous populations^(28)^. There are several interwoven factors that influence these strategies, including utilisation of Indigenous ways of knowing, engaging families in nutrition promotion and facilitating cultural continuity^(45,46)^. Indigenous communities have been known to possess a cultural connection to their land and identity which translates into their diets^(8)^. Thus, effective strategies to improve health outcomes require community-based participatory approach that supports incorporating Indigenous peoples’ cultural identity into the design^(47)^. The only study that utilised this process in this review found that measures targeted towards preventing overweight in toddlers reported a significant improvement in the intervention group^(39)^.

Evidence has shown that community participation in intervention design and implementation among Indigenous populations contribute to successful outcomes^(28)^. The positive association between Indigenous community engagement and health outcomes is well established, and it has also been shown that culturally adapted health interventions are more effective than traditional top-down approaches to health interventions^(28)^. Interventions led and developed by Indigenous communities, which include an understanding of Indigenous context and culture, are reported to have a higher chance of success^(45)^. In this review, only one study reported the inclusion of community participation in the design and implementation of the intervention^(39)^. This strong collaboration was reported as the main attributable factor to the high retention (86 %) and participation rates in the study. The acceptability of the intervention, measured by parents’ confidence to implement recommendations of the study, could also be attributed to community participation in the study and implementation. The review by Harding and Oetzel assessing the effectiveness of an implementation framework that provides a strong foundation for designing and implementing health interventions in Indigenous communities showed that there were high levels of community engagement in the studies^(28)^. The framework comprised of the following four elements: community engagement, a culture-centred approach, systems thinking and integrated knowledge translation. Of these four elements, lower levels of systems thinking and integrated knowledge translation were observed and were reported to likely inhibit the long-term sustainability and translation of evidence to practice. Although community participation was reported to contribute to participation rates in one of the studies in this review^(39)^, other studies^(38,40)^ also recorded improvements in nutrition and health outcomes without community participation in the study design. This could be attributed to the quality of the intervention as well as the mode of delivery as the interventions were delivered by trained health workers during home visits and face-to-face sessions. Regular monitoring and evaluation were performed, and nutritional guidance was provided to participants.

Studies have shown that intervention delivery is an important element of intervention among Indigenous populations^(30)^. None of the studies in this review reported the Indigenous status of the staff who delivered the intervention; thus, there is an uncertainty about the intervention being delivered by an Indigenous person(s). If the intervention was delivered by a non-Indigenous person(s), this could have attributed to the low breast-feeding implementation and duration rates recorded in the studies. Previous interventions that reported delivery by Indigenous programme workers have shown this to be effective^(48,49)^. The Strong Woman Strong Babies Strong Culture Program, which achieved some significant improvements in birth weights, was delivered by senior Aboriginal women^(48)^. This programme attributed connection and support for involvement in cultural events as an important factor of success. It also highlighted that Aboriginal knowledge and practice, including intervention delivery, was a fundamental component of the programme’s survival^(48)^. The Navajo Breastfeeding Intervention Program also reported that collaboration with locals who acted as cultural teachers, researchers and promoters was a major factor influencing the success of the programme^(49)^.

This review found a dearth of literature on the research subject addressing complementary food intake for Indigenous peoples. This is in keeping with the paucity of research on the topic more generally with Indigenous people as identified by Gwynn *et al*.^(31)^. While there were several studies that have researched the interventions among different groups of people across different regions globally, there were few studies that have investigated interventions to improve the nutrition and health of Indigenous children in the first 1000 d of life. The studies included in this review were conducted among only two Indigenous tribes in two countries, thus limiting the scope of this study. A strength of the Martinez *et al*. study was that it reported the nutritional status of the children prior to the intervention which provided a basis for effective comparison after the intervention.

A few additional limitations exist within this review that require acknowledgement. One limitation is that only studies published in English and with results published before June 2020 were included in this review. Thus, studies published in languages other than English and/or studies from ongoing interventions without published evaluations may have been missed. The dearth of studies available on the subject matter and a lack of high-quality studies was also a major limitation as they provide little evidence to guide practice.

In conclusion, there is need for more evidence to be added to this subject area in order to enhance the development of effective co-designed intervention processes among Indigenous communities. Outcome measures that could be considered by future studies include nutritional status of the population, dietary intake, breast-feeding intake and/or duration, and community acceptability of the intervention. Detailed description of the health and demographic status of the participants should also be considered.

Engaging Indigenous communities in the entire process of nutrition interventions for infants within their communities will have a range of beneficial effects by improving nutrition and health outcomes. The combination of Indigenous ways of knowing with empirical evidence will ensure that cultural sensitivity can be incorporated as the bedrock of nutritional interventions in Indigenous communities. As a result of this, future studies should endeavour to incorporate partnered approaches in the development of health interventions to ensure that Indigenous ways of knowing are led by community collaborations with researchers. When communities are engaged in interventions, it enables the integration of cultural knowledge and ensures that cultural values are upheld. Future research is needed to determine the effectiveness of including cultural context in the design of interventions for improving nutrition and health outcomes of children in the first 1000 d across Indigenous populations globally.

## Supplementary material

For supplementary material accompanying this paper visit https://doi.org/10.1017/S1368980021004328. click here to view supplementary material
